# Novel Iatrogenic Cause of Cholecystogastric Fistula

**DOI:** 10.7759/cureus.23531

**Published:** 2022-03-27

**Authors:** Andrew Alabd, Nikhita Dharbhamulla, Adam Elfant

**Affiliations:** 1 Medicine, Cooper University Hospital, Camden, USA; 2 Gastroenterology, Cooper University Hospital, Camden, USA

**Keywords:** esophagogastroduodenoscopy (egd), computed tomography (ct ), percutaneous cholecystostomy tube, cholecystogastric fistula, cholecystoenteric fistula

## Abstract

Cholecystogastric fistula (CGF) is a rare, abnormal communication between the gallbladder and the stomach that can be identified on abdominal computed tomography (CT) and confirmed via endoscopy. CGFs are not usually problematic. However, they can cause fatal complications. We present a case of an adult patient with a history of percutaneous cholecystostomy (PC) presenting with non-specific gastrointestinal (GI) symptoms and found to have an iatrogenic CGF. The fistula is believed to be secondary to the PC tube. CGF from PC has not been described in the literature before.

## Introduction

Cholecystoenteric fistula (CEF) is an abnormal communication between the gallbladder and luminal gastrointestinal (GI) tract. Cholecystoduodenal fistula (CDF) is the most common, followed by cholecystocolonic fistula (CCF) and rarely cholecystogastric fistula (CGF) [1]. There have been multiple reports of CGF as far back as 1956 [2]. While CGF is usually associated with chronic cholecystitis or long-standing cholelithiasis, other reported causes include peptic ulcer disease, inflammatory bowel disease, and GI malignancy [1,3]. We present a case of a percutaneous cholecystostomy (PC) tube causing CGF. Our case is unique in that it is the first iatrogenic case of CGF described in the literature as a possible complication of a PC tube. This article was previously presented as a meeting abstract at the 2020 American College of Gastroenterology (ACG) Annual Scientific Meeting on October 23, 2020.

## Case presentation

A 77-year-old man had a past medical history of coronary artery disease with subsequent stent placement, heart failure, and cholecystitis followed by PC one year prior to presenting with CGF. The patient presented to our institution with a five-day history of nausea and non-bloody bilious emesis. The cholecystostomy tube had been removed one month earlier due to tube dislodgement. He had no prior history of malignancy, radiotherapy, or inflammatory bowel disease.

On arrival, the patient was hemodynamically stable with no fever or leukocytosis. Physical examination revealed a soft, non-tender, and non-distended abdomen with normal bowel sounds, negative Murphy’s sign, and the presence of a wound in the right upper quadrant (RUQ) with a small amount of bilious drainage.

**Figure 1:**
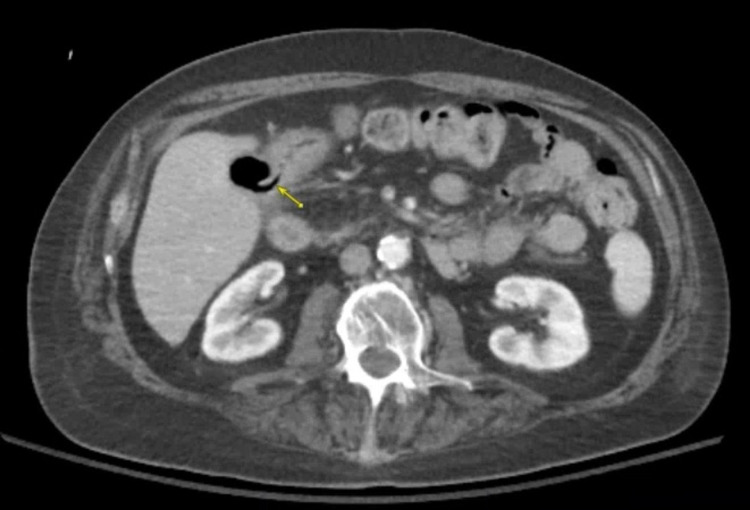
Computed tomography scan showing liner gas-filled tract (arrow) between the stomach and gall bladder, consistent with cholecystogastric fistula.

**Figure 2:**
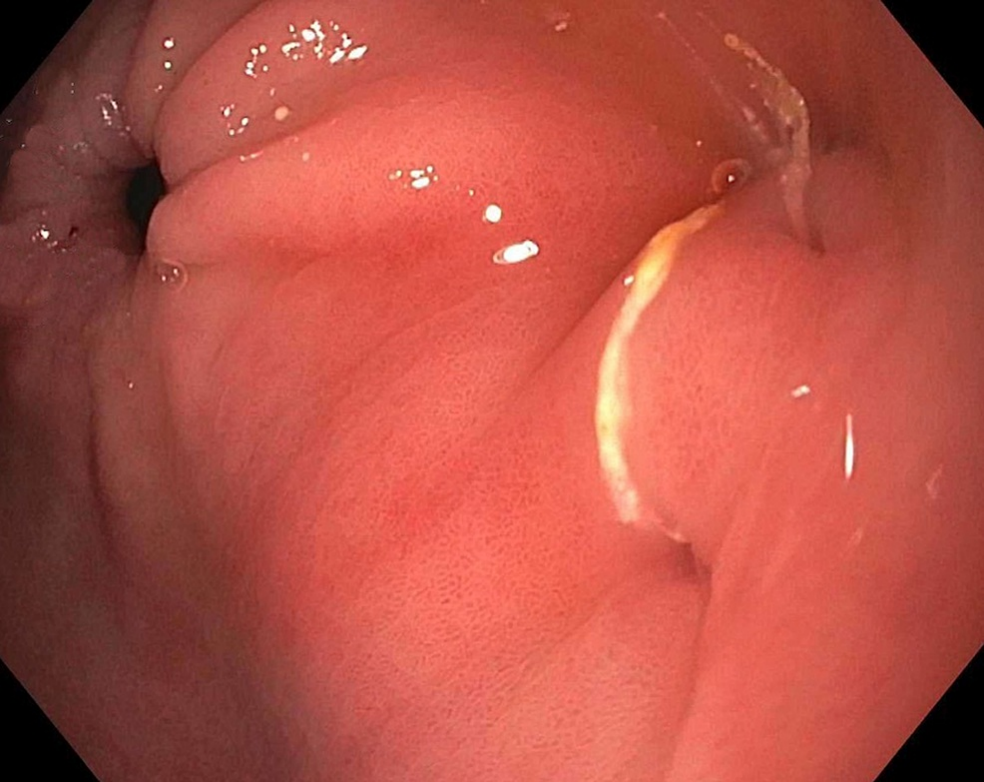
Esophagogastroduodenoscopy showing draining fistula within the gastric antrum.

Computed tomography (CT) scan showed intraluminal gas within the gallbladder with a linear gas-filled tract extending into the gastric antrum, compatible with a CGF, and no gallstones were visualized (Figure 1). Esophagogastroduodenoscopy (EGD) showed evidence of a fistula in the gastric antrum with drainage (Figure 2). The patient was considered to be too high risk for a fistula repair via surgical intervention and no further interventions were undertaken. His symptoms improved with supportive care and he was safely discharged in stable condition.

## Discussion

Clinical presentation of CGF varies from clinically silent and asymptomatic fistula with no complications to gall stone ileus, cholangitis, GI hemorrhage, or gastric outlet obstruction [4,5].

Unlike percutaneous transhepatic cholangiography, endoscopic retrograde cholangiopancreatography, or magnetic resonance cholangiopancreatography, CT imaging is less invasive and is a routine and readily available modality to visualize CGF [6,7]. This made it the appropriate modality of choice for diagnosis for our patient. CGF appears as a slight deficiency of the gall bladder wall on CT imaging [7] and occasionally with close adherence of the gastric antrum wall and gall bladder wall [8]. In this case, the CT imaging showed a linear gas-filled tract extending from the gall bladder into the gastric antrum.

With the advancement in radiological and endoscopic modalities, early diagnosis and proper management can be safely achieved. Endoscopy can be both diagnostic and therapeutic in CGF [9]. EGD findings of CGF include a small fistulous opening in the stomach antrum with pus exuding through the opening [9]. When CGF is secondary to a stone, endoscopic retrieval of the stone as treatment of CGF offers a safer and more prudent solution. Endoscopic lithotomy or lithotripsy should always be considered before surgery [10]. Other interventions include mechanical, electrohydraulic, or laser lithotripsy, or surgical intervention with possible fistula repair [7,11].

Given the absence of cholelithiasis, neoplasia on imaging, or other etiology that can explain the fistula formation in our patient, we hypothesize that CGF was secondary to the cholecystostomy tube. PC tubes have not been reported previously as an etiology of CGF in the literature. Patients with significant comorbidities and poor functional status can be managed conservatively [5].

## Conclusions

CGF, though rare, carries significant morbidity and mortality. Thus, it has to be kept as one of the differentials in patients presenting with non-specific GI symptoms and recent PC tube. Patients with significant comorbidities and poor functional status can be managed conservatively, as in the case of our patient. In our patient’s case, the fistula was likely secondary to the cholecystostomy tube given the absence of other etiology that can explain the fistula formation.
